# Quiescin Sulfhydryl Oxidase 1 (QSOX1) Secreted by Lung Cancer Cells Promotes Cancer Metastasis

**DOI:** 10.3390/ijms19103213

**Published:** 2018-10-17

**Authors:** Hye-Jin Sung, Jung-Mo Ahn, Yeon-Hee Yoon, Sang-Su Na, Young-Jin Choi, Yong-In Kim, Soo-Youn Lee, Eung-Bae Lee, Sukki Cho, Je-Yoel Cho

**Affiliations:** 1 Department of Biochemistry, BK21 PLUS Program for Creative Veterinary Science Research and Research Institute for Veterinary Science, College of Veterinary Medicine, Seoul National University, Seoul 08826, Korea; yuki0407@hanmail.net (H.-J.S.); efrobert@hanmail.net (J.-M.A.); yhyoon1@knu.ac.kr (Y.-H.Y.); deshitart@naver.com (S.-S.N.); jin2627@gmail.com (Y.-J.C.); vaioman@snu.ac.kr (Y.-I.K.); 2 Departments of Laboratory Medicine & Genetics and Medicine, Samsung Medical Center, Sungkyunkwan University School of Medicine, Seoul 06351, Korea; suddenbz@skku.edu; 3 Department of Thoracic and Cardiovascular Surgery, Kyungpook National University Medical Center, Daegu 41944, Korea; eungbae@gmail.com; 4 Department of Thoracic and Cardiovascular Surgery, Seoul National University Bundang Hospital, Seoungnam-si, Gyeonggi-do 13620, Korea; skcho@snubh.org

**Keywords:** lung cancer, biomarker, proteomics, QSOX1, secretome

## Abstract

As lung cancer shows the highest mortality in cancer-related death, serum biomarkers are demanded for lung cancer diagnosis and its treatment. To discover lung cancer protein biomarkers, secreted proteins from primary cultured lung cancer and adjacent normal tissues from patients were subjected to LC/MS–MS proteomic analysis. Quiescin sulfhydryl oxidase (QSOX1) was selected as a biomarker candidate from the enriched proteins in the secretion of lung cancer cells. QSOX1 levels were higher in 82% (51 of 62 tissues) of lung cancer tissues compared to adjacent normal tissues. Importantly, QSOX1 serum levels were significantly higher in cancer patients (*p* < 0.05, Area Under curve (AUC) = 0.89) when measured by multiple reaction monitoring (MRM). Higher levels of QSOX1 were also uniquely detected in lung cancer tissues, among several other solid cancers, by immunohistochemistry. QSOX1-knock-downed Lewis lung cancer (LLC) cells were less viable from oxidative stress and reduced migration and invasion. In addition, LLC mouse models with QSOX1 knock-down also proved that QSOX1 functions in promoting cancer metastasis. In conclusion, QSOX1 might be a lung cancer tissue-derived biomarker and be involved in the promotion of lung cancers, and thus can be a therapeutic target for lung cancers.

## 1. Introduction

Lung cancer has been the leading cause of worldwide cancer-associated mortality. Recent 2018 cancer statistics show that lung cancer, along with pancreatic and stomach cancer, shows only minor increase in the 5-year survival rate among all cancers [1,2]. It is speculated that the low survival rate of lung cancer is largely due to the low diagnostic rate in the early stages. Advances of imaging technology have also been improved to detect smaller lesions than before. However, for economic burden, exposure to radiation and still high rate of false-positive rate, other supportive types of diagnosis methods are still required in lung cancer diagnosis.

Protein biomarkers are known to represent the status of body better than other types of biomarker molecules, such as DNA. Along with advances in proteomics, protein biomarker discovery and clinical application have been actively studied and are regarded as a clinically valuable study by many groups [3,4]. Several protein biomarkers for lung cancer are being developed, including cytokeratin-19 fragments (CYFRA 21-1) [5,6], carcinoembryonic antigen (CEA) [7], neuron-specific enolase (NSE) [8], and cancer antigen-125 (CA-125) [9]. However, the challenges in biomarker discovery and the time-consuming process leading up to clinical application hinder the development of biomarkers for clinical uses [10].

Lung cancer biomarker development is facing similar problems to other cancer biomarkers. First, blood-derived protein biomarkers have a tendency to show cross-reactivity with other types of cancers. Second, selecting final biomarkers to be developed for clinical applications should be preceded by large-scale validation [11]. Third, monoclonal antibodies are essential in developing immunoassay-based diagnostics to be used in the clinics. Due to these hurdles, although hundreds of biomarkers have been discovered and reported by many research groups, few are in clinical use at present.

The secretome refers to all sets of molecules secreted by a living cell, a tissue, or an organism through any mechanism. Secreted proteins have been proposed as a new source of biomarker discovery [12,13]. The secretome could be obtained from several different sources. Fora broad scope, serum or plasma can be considered as a kind of secretome. For the secretome in more proximal locations, tissue effusions, such as ascites or pleural effusions, might contain abundant secretions. The most proximal secretome to cancers would be the one collected from cancer cell cultures. Conditioned media (CM) of cells have been suggested as a complementary source of biomarker discovery [14]. It is expected that biomarker discovery from more proximal fluid could reveal more disease- and cell-type-specific biomarkers.

In this study, to discover lung cancer specific protein biomarkers, proteomic analysis of secreted proteins from primary cultured lung cancer cells was conducted. One selected biomarker candidate QSOX1 has been further verified in tissues and elevation of QSOX1 in the serum has been validated by MRM analysis. The role of QSOX1 in cancer progression was also assessed by knock-down of QSOX1 in LLC cells in vitro and in vivo. The results indicated that elevated QSOX1 might be a lung cancer selective biomarker and be involved in the metastasis or progression of lung cancers.

## 2. Results

### 2.1. Strategy for Lung Cancer Biomarker Discovery from the Secretome of Primary Lung Cancer Cells

**Figure 1:**
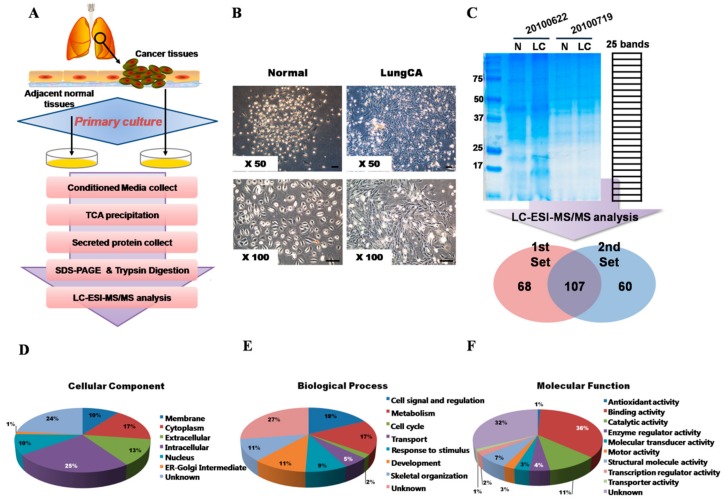
MS/MS analysis of secreted proteins and GO analysis of the identified proteins. (**A**) Schematic diagram of experimental process from primary culture of lung cancer tissues, to secretome enrichment, and LC–MS/MS analysis of secretome. (**B**) Cells isolated by collagenase treatment from the lung cancer tissues and their adjacent corresponding normal tissues. Isolated cells from cancer tissues and corresponding normal tissues show different cellular morphologies. Scale bar, 100 µm. (**C**) Secreted proteins, enriched by conditioned media, from primary cultured cells of two different lung cancer patients and separated by 1-DE-SDS-PAGE. Separated proteins were divided into 25 bands and subjected to in-gel digestion. Tryptic peptides were subjected to LC–ESI–MS/MS analysis. MS analysis discovered 107 common proteins detected in both MS analytical sets, and 68 were subjected to further GO analysis. (**D**) Identified proteins analyzed by their cellular component, (**E**) biological process, and (**F**) molecular functions. N: normal, LC: lung cancer.

To discover lung cancer-selective proteins, a secretome-based lung cancer biomarker discovery strategy was applied (Figure 1A). For the collection of secreted proteins from the tissues of lung cancer and adjacent normal tissues, cells from the tissues freshly acquired from the patients on surgery were separated and cultured. Normal and cancerous epithelial cells were not isolated separately but cultured, together with stroma-like cells, to mimic cell-to-cell interactions in in vivo environments. Cells isolated from the adjacent normal tissues showed typical cobble stone-like morphologies of epithelium and cells from lung cancer tissues were irregular and showed a mesenchymal- or fibroblast-like morphology (Figure 1B).

### 2.2. LC–ESI–MS/MS Analysis of Secretome and Biomarker Candidate Discovery

Lung cancer tissues and their corresponding normal tissues from two lung cancer patients, who underwent surgery (#20100622 and #20100719), were used for cell culture establishment. The same amount of secreted proteins, enriched by the CM, of the normal and lung cancer-derived cells were separated by 1D-SDS-PAGE. To identify biomarker candidate proteins, gels were sliced into 25 bands. In-gel tryptic-digested peptides were analyzed by LC–ESI–MS/MS. From the first and second MS analyses, 175 and 167 proteins were identified, respectively, and 107 proteins appeared to be in common (Figure 1C). Identified proteins were analyzed in their cellular components, biological processes and molecular functions (Figure 1D–F). As expected from several previous reports, the proteins from the CM appeared to include many intracellular protein contaminants (approximately 25% of the identified proteins) from the apoptotic cell debris caused by the serum-deprived culture condition [15,16,17] However, cell membrane or extracellular matrix proteins also constituted about 23% of the total proteins identified.

For the selection of biomarker candidates showing significant difference between adjacent normal and cancer, relative peptide hits after normalization were counted by total ion current (TIC) of each mass run. Protein levels were evaluated by the value of fold change calculated by the relative peptide hit of the lung cancer set divided by that of normal tissue set. Proteins showing the fold change over 1.5 or under 0.67 in both individuals were selected. Fifteen out of 135 proteins showed significant increases in lung cancer compared to normal, and 4 proteins showed significant decreases (Table 1). To find secreted protein biomarker candidates from the up- and down-regulated candidates, we selected candidates, which included extracellular matrix protein in their GO analysis and had signal peptides in the amino acid sequences.

### 2.3. Elevation of QSOX1 in Lung Cancer Tissues Compared to Adjacent Normal Tissues

**Figure 2:**
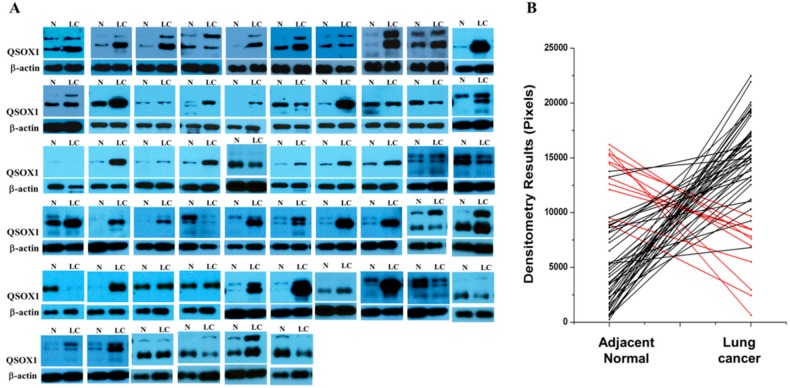
QSOX1 proteins present at higher levels in lung cancer tissues compared to corresponding adjacent normal tissues. (**A**) Proteins extracted from lung cancer tissues and their corresponding tissues from 56 lung cancer patients. For each sample, 2.5 μg of proteins were subjected to Western blot analysis. QSOX1 was detected as two forms, long (74 kDa) and short secreted forms (65 kDa). (**B**) Graphs of densitometry analysis data showed 48 out of 56 patients (about 85.7%) had an increase in QSOX1 protein expression in lung cancer tissue, compared to adjacent normal tissue. N: normal, LC: lung cancer.

Among the proteins with significant change, QSOX1 was selected as a lung cancer tissue biomarker candidate for further validation. In mass spectrometry analysis, four and two unique peptides of QSOX1 appeared in samples #20100622 and #20100719, respectively. Spectral counts of total peptides of normal and lung cancer samples after normalization were 5.5 and 9.1, and 1.0 and 4.3, respectively, in each patient (Table S1, Supplementary Materials). To verify QSOX1 levels in lung tissues, Western blot analysis of QSOX1 in lung cancer patient tissues was carried out. Proteins were extracted from lung cancer tissues and their corresponding normal tissues from 56 lung cancer patients (Figure 2A). The Western blot data were quantified by densitometric analysis and the graph shows that 48 patients out of 56 (82.2%) showed an increase of QSOX1 protein levels in the lung cancer tissues, in comparison to adjacent normal tissues (Figure 2B).

To validate the presence of QSOX1 in lung cancers, a mouse model carrying LLC cells was established. Mice were divided into 4 groups, intravenously injected with LLC cells or PBS, and sacrificed at two different time points; 10 days and 35 days (Figure S1A, Supplementary Materials). Proteins were extracted from lungs without nodules and adjacent normal lung tissues of mice with lung cancer, lung cancer nodules and metastasized nodules. QSOX1 expression was tested in each sample. In accordance with human cases, lung cancer tissues from the mice showed elevated QSOX1 levels. In addition, metastasized nodules in other organs also showed higher QSOX1 protein levels (Figure S1B, Supplementary Materials). These results suggested that QSOX1 might be involved in the metastasis of lung cancers.

### 2.4. Increased Expression of QSOX1 Preferential in Lung Cancer Tissues

**Figure 3:**
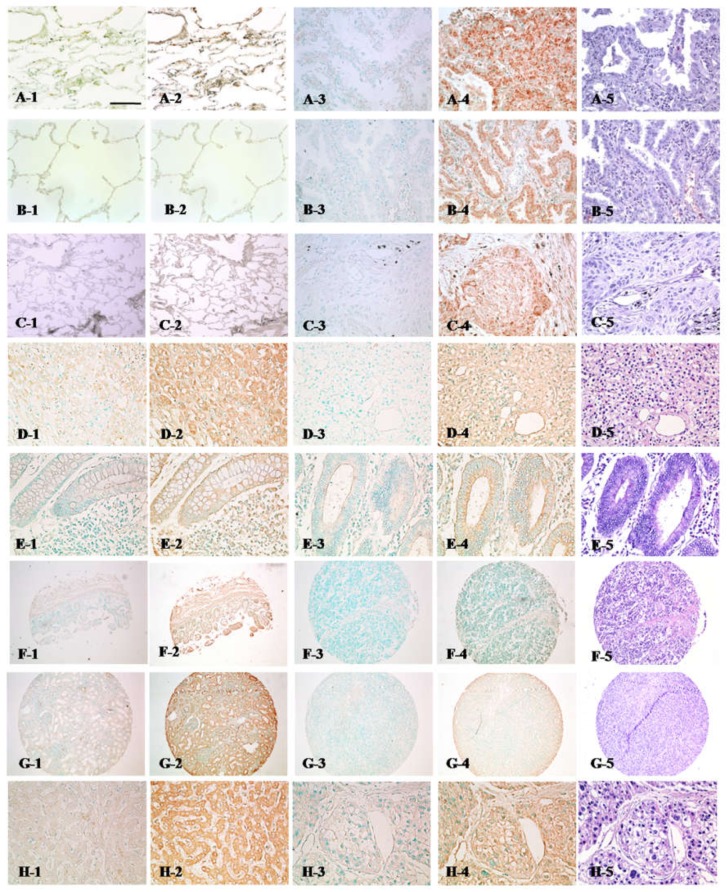
Immunohistochemical staining of QSOX1 in various cancer tissues and their corresponding normal tissues. QSOX1 protein expressions in lung cancer tissues were confirmed by immunohistochemistry on tissue microarray slides. Column 1 is the slides stained without antibody for corresponding normal tissues of column 2 (negative control); column 2, stained with anti-QSOX1 antibody for each organ; column 3, stained without antibody (negative control) for column 4; column 4, stained with anti-QSOX1 antibody on cancer tissues for each organ. Column 5 is H&E staining of column 4 at the next serial section. Scale bar, 100 µm. (**A**,**B**) Lung adenocarcinoma, (**C**) squamous cell lung cancer, (**D**) pheochromocytoma in adrenal gland, (**E**) adenocarcinoma in colon, (**F**) large cell lymphoma in ileum, (**G**) renal cell carcinoma, and (**H**) hepatocellular carcinoma.

From the results of the Western blot verification in lung cancer tissues, it can be confirmed that QSOX1 is up-regulated in lung cancer tissues. To further prove QSOX1 levels in tissues, an immunohistochemical analysis was conducted on tissue microarray (TMA) slides of various cancer tissues with corresponding normal tissues. From the results of the immunohistochemical analysis, high levels of QSOX1 protein were detected in lung cancer cells. Regardless of histological types, adenocarcinoma or squamous lung carcinoma, QSOX1 over-expression in lung cancer tissues was confirmed (Figure 3A–C). However, no significant increase of QSOX1 was detected in pheochromocytoma of adrenal gland, colon adenocarcinoma, large cell lymphoma of ileum, renal cell carcinoma, or hepatocellular carcinoma (Figure 3D–H), although a few normal tissues such as liver, colon and kidney showed high expressions of QSOX1 (Figure 3(H-2,E-2,G-2)). From the results, it is presumed that the QSOX1 increase in lung cancer is not the common cancer response, but, to some extent, lung cancer-unique.

### 2.5. Validation of QSOX1 in the Sera of Lung Cancer Patients by MRM Analysis

So far, we have shown that lung cancer tissues had higher levels of QSOX1 proteins, of which levels were somewhat lung cancer-unique compared to other cancers tested. To confirm whether the increase of QSOX1 in lung cancer tissues can also be detected in the sera of lung cancer patients, a MRM protocol for QSOX1 measurement was developed. Due to the low concentration of QSOX1 in the serum, MRM was adopted to measure QSOX1 in the sera depleted of the two most abundant components, albumin and IgG. Among several QSOX1 peptides recommended by the Skyline program, VGSPNAAVLWLWLWSSHNR was finally selected based on the results of standard MRM of a few samples. The *m*/*z* of precursor ions, transitions, and collision energy for the selected QSOX1 peptide were optimized (Table S2, Supplementary Materials). Detailed area measurements and quantitation procedures are described in Supplementary Materials.

**Figure 4:**
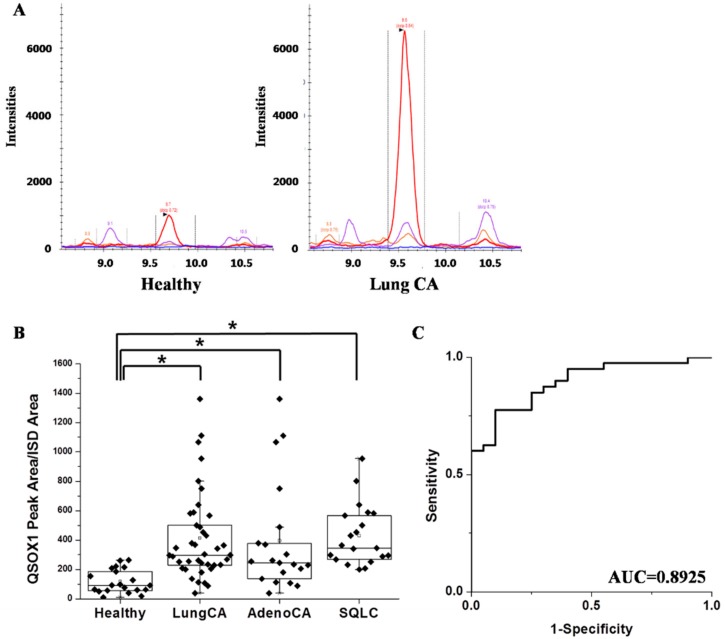
QSOX1 proteins were more abundant in lung cancer patients’ sera compared to those in healthy controls. (**A**) Representative transition peak pattern of QSOX1 peptide, VGSPNAAVLWLWLWSSHNR, in healthy controls and lung cancer patients. (**B**) Box plot showing the normalized measured area of QSOX1 peptide in 20 healthy individuals, 20 adenocarcinoma patients (AdenoCA), and 20 squamous lung cancer patients (SQLC). (**C**) ROC plotted for 60 individuals with the area under curve (AUC) value of 0.8925. (* denotes *p* < 0.05, compared to healthy individual samples in the analysis of significant variance).

Samples of 20 healthy controls, and 40 lung cancer patients, including 20 lung adenocarcinoma and 20 squamous lung cancer patients, were subjected to the MRM analysis. Three mass runs were serially conducted for each sample. Representative peaks of a healthy control and a lung cancer of each peptide are shown in Figure 4A. Higher transition peaks with larger peak areas were detected in lung cancer samples compared to those in the healthy control for both peptides. Although in the Immunohistochemical analysis (IHC) results, a few organs with normal tissues showed high QSOX1 expressions, none of the serum from healthy individuals showed high QSOX1 levels and there was a statistically significant difference between healthy controls and lung cancer groups (Figure 4B). The diagnostic ability has been validated by Receiver Operating Characteristic (ROC) curves. The Area Under curve (AUC) value of QSOX1 peptide was 0.8925 (Figure 4C). The QSOX1 peptide turned out to have a high diagnostic value for distinguishing lung cancer from healthy individuals in serum.

### 2.6. QSOX1 Knock-Down Decreases Lung Cancer Metastasis

**Figure 5:**
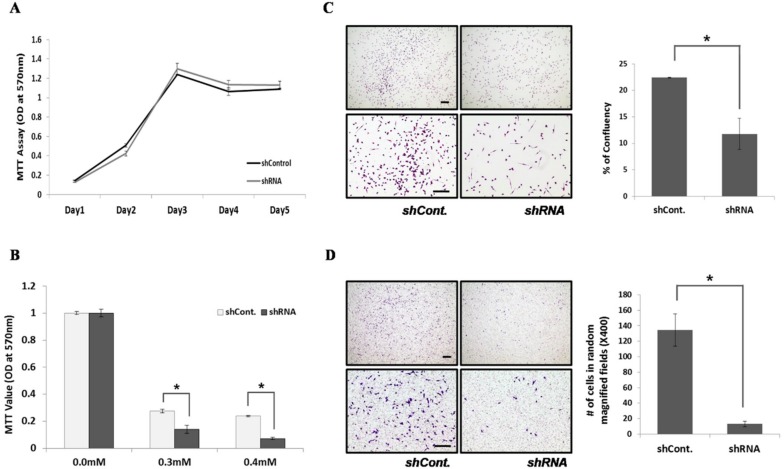
QSOX1 expression in lung cancer cells promotes cancer progression by anti-apoptotic, pro-migration and pro-invasion functions. (**A**) Cell proliferation of LLC transfected with shRNA control (shCont) or QSOX1 shRNA (shQSOX1) measured by MTT assay. (**B**) Cell survival from oxidative stress measured by MTT assays in various H_2_O_2_ concentration conditions. (**C**) Using a Boyden chamber, the cell migration assay was carried out and cells at the lower chamber was stained and counted. (**D**) Using a Boyden chamber with the basement membrane Matrigel coated, the cell invasion assay was carried out and cells at the lower chamber was stained and counted. Scale bar, 100 µm.

The LLC lung cancer mouse model experiment showed the high expression of QSOX1 in metastasized lung cancers. From this result, we hypothesized that QSOX1 is involved in lung cancer metastasis. First, to check whether QSOX1 affects cancer initiation by promoting cell proliferation, cell proliferation of control cells and QSOX1-knock-downed cells by shRNA was compared. There was not much difference between these two cells in proliferation rates (Figure 5A). Second, we tested whether QSOX1 could help cancer cells survive in oxidative stress. The viability of LLC cells with knock-downed QSOX1 was more sensitive to H_2_O_2_ treatment oxidative stress conditions and thus significantly decreased in knock-downed cells (Figure 5B). Based on this result and the reports of Shi et al. [18], it can be deduced that QSOX1 secreted from cancer cells might protect cancer cells in the tumor mass from apoptosis in the oxidative stress condition, occurring as the tumor volume grows larger. It is of note that QSOX1 secreted from fibroblast functions in regulating the extracellular matrix [19]. To test extracellular matrix (ECM)-modulating functions of QSOX1 secreted by lung cancer cells, LLC cells were subjected to migration and invasion assays. In the migration assay, the QSOX1-knock-downed LLC cells showed a less migration rate than control cells (Figure 5C). Using a Boyden chamber coated with basement membrane Matrigel, the cell invasion assay showed that QSOX1-knock-downed cells had a greatly decreased invasiveness compared to control cells (Figure 5D).

**Figure 6:**
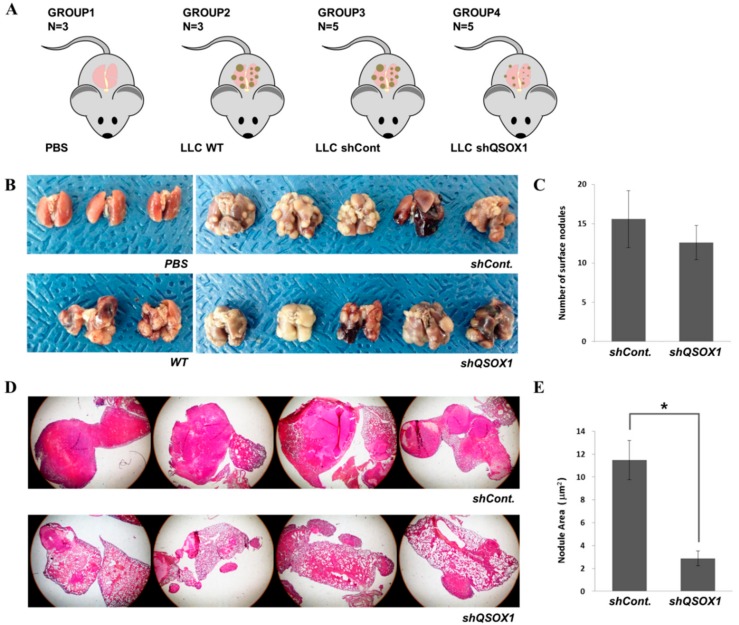
QSOX1 expression in lung cancer cells promotes cancer progression in vivo. (**A**) Mice in four groups, intravenously injected with PBS, wild-type LLC, LLC with shCont and LLC with shQSOX1, respectively. (**B**) All of the mice injected with LLC cells (groups 2–4) had lung cancer nodules on the lung, but with different severities. (**C**) The number of surface nodules that was counted shows no significant difference between groups 3 and 4. (**D**) Slides of the lungs stained with H&E. (**E**) All nodule areas calculated and combined for each mouse show significant decrease in the shQSOX1 (group 4) compared to shCont (group 3). (* indicates *p* < 0.05 in the analysis of significant variance).

To confirm the QSOX1 contribution to lung cancer metastasis in vivo, invasiveness of QSOX1-knock-downed cells was evaluated in the LLC mouse model. Mice were grouped into four categories; they were injected intravenously with either PBS, wild-type LLC cells (LLC WT), LLC with control shRNA (LLC shCont), and LLC with shQSOX1 (LLC shQSOX1) (Figure 6A). Mice of groups 2, 3 and 4 had lung cancer nodules 35 days after injection, and the surface nodule number was counted. The nodule numbers were not much different between LLC shCont and LLC shQSOX1 groups (Figure 6B,C). However, the nodule sizes of the shQSOX1 group were significantly smaller than the nodules of the LLC shCont group (Figure 6D). When the area of nodules was measured, the tumors in the QSOX1-knock-downed group were significantly smaller than those in the shCont group (Figure 6E). From these results, it can be inferred that QSOX1 might not be involved in cancer initiation but does contribute to cancer progression, such as tumor invasion and metastasis.

## 3. Discussion

In this study, we identified lung cancer-secreted protein QSOX1 as a lung cancer-selective biomarker candidate with a proteomics approach and validated its increase in tissues and serum of lung cancer patients. From the results of the immunohistochemical analysis and LLC mouse model, the increase of QSOX1 in lung cancer cells has been confirmed. The involvement of QSOX1 in cancer progression has been validated in in vitro and in vivo experiments with the QSOX1-knock-downed LLC lung cancer cell line.

### 3.1. QSOX1, Lung Cancer Tissue-Selective Biomarker

Along with the development of proteomic technology, protein biomarkers for the lung cancer diagnosis have also been reported. Many researchers have been conducted to identify numerous relevant proteins in various diseases, including cancers through mass spectrometry-based clinical proteomics. Although advances in mass spectrometric technologies have made it possible to identify over 50,000 unique peptides covering almost 5000 proteins, there are still limitations to uncovering unique proteins.

Cancers are also considered a chronic inflammatory disease and show similar inflammatory responses [20,21,22]. For this reason, several acute phase proteins have been reported as lung cancer biomarkers. We have also previously reported certain acute-phase proteins as lung cancer biomarkers: serum amyloid A (SAA) [23], haptoglobin (Hp) β chain [24] and complement 9 (C9) [25]. The elevation of acute-phase proteins in the body fluid is suspected to be resulted from the secretion of the major acute-phase protein-secreting organ, the liver, by the inflammatory signals released by lung cancers. For this reason, serum biomarkers are likely to show cross-reactivity to other types of solid cancers, which could show similar systemic responses.

Study of the secretome of cancer cells has been suggested as an alternative source of biomarker discovery for tissue-selective biomarkers. Several studies of the lung cancer secretome have been conducted. Lung cancer cell lines have been subjected to these studies, but not many studies have been conducted on primary lung cancer cells or organ cultures [26]. Studies on the secretome of about 15 different lung cancer cell lines have been reported. According to the studies, new proteins have been reported, which have not been discovered in the serum as lung cancer biomarkers. These biomarker candidates include Cathepsim-D [27], L-lactate dehydrogenase B chain (LDHB) [28], translationally controlled tumor protein (TCTP) [29], triose phosphate isomerase (TPI) [29], dihydrodiol dehydrogenase (DDH) [30], etc. However, QSOX1 has never been discovered in the secretome analysis of lung cancer cell lines.

The main limitation of cell secretome study is that two-dimensional cell culture cannot fully mimic complex cancer microenvironments [14]. To partially overcome these concerns, stromal supporting cells isolated from the tissues were cultured together without any sorting. Therefore, the cancer cells were maintained with other different heterogeneous cells that included different types of immune cells infiltrated in the cancer tissues and fibroblasts. From the Western blot analysis of the tissue protein, we confirmed the increase of QSOX1, and the immunohistochemical analysis of tissue microarray revealed that QSOX1 expression was increased in the lung cancer tissues.

### 3.2. Establishment of MRM for the Measurement of QSOX1 in Sera

MRM or selected reaction monitoring (SRM) are mass spectrometry-based protein/peptide quantification methods [31,32]. MRM depends on mass spectrometry and its current limit of quantitation (LOQ) is at the low attomole level [33,34]. The strength of this technique is that it is one of the methods best suited to the validation of hundreds of protein biomarkers in a large number of samples. Compared to the conventional clinical methods, such as ELISA, MRM shows highly correlated data and even better results when the target shows saturated results in ELISA [35].

To detect serum proteins at lower concentrations, stable isotope standards and capture by anti-peptide antibodies (SISCAPA) [33,36] and the depletion of abundant proteins have been combined in MRM. To overcome the wide range of serum proteins, abundant protein depletion in the sample preparation step should precede MRM analysis. This step increases the chance of detecting proteins at lower concentrations in the sera. Following the depletion of abundant albumin and IgG in the serum, the optimal condition of the QSOX1 MRM analysis method has been established. QSOX1 was detected in the sera of lung cancer patients, and although the level was very low, lung cancer patients showed higher levels of serum QSOX1 compared to healthy individuals. The AUC curve for differential diagnostics was close to 0.9. This implies that QSOX1 increase in the sera can be a more selective lung cancer biomarker than blood-born biomarkers, of which most show high cross-reactivity to other types of solid cancers.

### 3.3. QSOX1 Plays a Role in Cancer Progression and Metastasis

QSOX1 had first been reported to be expressed in human lung fibroblast. QSOX1 expression was increased as the cells reached high confluence, suggesting that it might have a role in induction and maintenance of the quiescent state of cells [37]. Since the first report of the QSOX1 protein, enzymological studies of the protein have been conducted [38,39,40]. It is reported that QSOX1 catalyzes the generation of disulfides as part of the Erv (Endogenous retroviruses) family by reducing oxygen to hydrogen peroxide. The tissue expressions of QSOX1 have also been reported in several studies. In breast cancer, it is reported that high expression of QSOX1 leads to the reduction of tumorigenesis and correlates to the prognosis of the patients [41]. In pancreatic cancer, association between QSOX1 and cancer metastasis has also been studied [42]. However, QSOX1 has never been reported in lung cancer and its biological function in cancer progression has never been thoroughly studied. From a recent study, it has been suggested that QSOX1 secreted from the fibroblast modulates the extracellular matrix by enzymatic incorporation of laminin [19]. According to the previous studies and our results of the knock-down experiment of the QSOX1 in vitro, it is suggested that QSOX1 secreted from the cancer cells promotes cancer metastasis by modulating the extracellular matrix, as it does in fibroblasts. In accordance with previous studies, our results showed that QSOX1 can promote cancer progression by promoting cancer metastasis in vitro and in vivo. It is also reported that inhibition of enzymatic activity of QSOX1 suppressed invasion of pancreatic and renal cancer cell lines [43] and QSOX1 inhibited the autophagic reflux [44]. Inferred from these two published data and our results, it is suggested that QSOX1 might be a therapeutic target for lung cancer.

## 4. Materials and Methods

### 4.1. Human Tissues and Serum Samples

Tissue and serum samples were obtained from patients at Seoul National University Bundang Hospital (IRB# B-1201/143-003.), and Samsung Medical Center (IRB No. 2008-06-007-005). Informed consent was obtained from the tissue donors. Serum was separated from whole blood by centrifugation within 4 h after collection and was stored at −70 °C until use. Tissues were obtained during the surgery and delivered in the RPMI media with antibiotics for the primary culture. For storage, the tissues were rapidly cooled by liquid nitrogen. The information of patients who provided the samples used in the experiments is summarized in Table S3 (Supplementary Materials).

### 4.2. Cell Culture

#### 4.2.1. Primary Culture of Lung Tissues

Primary culture of lung pneumocytes was carried out according to modified protocols of a previously reported study [45]. Lung tissues freshly collected in DMEM media were chopped with blades and treated with collagenase Type I (0.5 mg/mL) in a 37 °C shaking incubator for 1.5 h. Tissue clumps were removed by filtration with mesh, and isolated cells were plated on culture dishes and cultured in media containing 10% fetal bovine serum for 4 days. Then, dead or unattached cells were washed away. After one passage of subculture for the amplification, the same number of cells was seeded on the plate for the CM collection. Normal and cancerous epithelial cells were not isolated separately but cultured together with stromal cell types, to mimic cell-to-cell interactions in in vivo environments.

#### 4.2.2. Cell Line Culture

LLC cells were cultured in Dulbecco’s Modified Eagle’s Medium (DMEM/High glucose) (Hyclone, South Logan, UT, USA) containing 10% fetal bovine serum (Hyclone) and 1% antibiotic-antimycotics (GIBCO, Grand Island, NY, USA).

### 4.3. Secretome Collection and Protein Precipitation

The same number of cells derived from lung cancer tissues and adjacent normal tissues were plated on 100 mm dishes. After 24 h of culturing in 10% FBS-supplemented DMEM, having reached 60–70% of cell confluency, the media was changed to serum-free media to collect CM containing secretome. To remove contaminants from FBS, cells were washed with pre-warmed PBS four times and with pre-warmed serum free media two times before media change. After 24 h, CM was harvested [17,46]. To remove cell debris, collected media was centrifuged at 2000 rpm, for 10 min and the supernatant was further filtered with a 0.2 μm pore filter. Secreted proteins were enriched from the filtrated CM by the TCA (Trichloroacetic acid) purification method. In brief, 1 volume of 100% (*w*/*v*) TCA (Sigma Aldrich, St. Louis, MO, USA) was added to 4 volumes of media, then incubated for 4 h at 4 °C and centrifuged at 13,000 rpm, for 30 min. The supernatant was removed, leaving the protein pellet intact. The pellet was washed with cold acetone and then re-dissolved in RIPA buffer (Thermo Scientific, Waltham, MA, USA) with protease inhibitor cocktail (Roche, Indianapolis, IN, USA). The protein concentration was measured by Bradford assay (Bio-Rad, Berkeley, CA, USA).

### 4.4. Proteomic Analysis of Secretome Protein

Following one-dimensional gel electrophoresis and Coomassie staining, gels were excised and subjected to in-gel trypsin digestion, as previously described [47]. Briefly, after destaining in 75 mM of a mixture with an ammonium bicarbonate/40% ethanol volume ratio of 1:1, gels were subjected to both reducing (5 nM DTT in 25 mM ammonium bicarbonate) and alkylating (55 mM iodoacetamide) conditions, each at room temperature for 30 min. After dehydration with ACN (20 µg/mL), sequencing grade trypsin (Roche Applied Science) containing 25 mM ammonium bicarbonate was added and the slice was incubated at 37 °C, overnight. Tryptic peptides were eluted with 0.1% formic acid.

LC–MS/MS analysis was performed using Thermo Finnigan ProteomeX work station LTQ linear IT MS (Thermo Electron, San Jose, CA, USA) equipped with NSI source (San Jose, CA, USA). Analysis conditions for mass spectrometry were the same as previously reported [25]. MS/MS data were searched based on the IPI human protein database (version 3.29) using the SEQUEST algorithm (Thermo Electron). Scaffold ver. 01_07_00 was used to validate MS/MS-based peptides and protein identification.

### 4.5. Knock-Down of QSOX1 and In Vitro Analysis

#### 4.5.1. Knock-Down of QSOX1

The shControl vector and shQSOX1 in retroviral vectors were purchased from Origene, Rockville, MD, USA. LLC cells were transfected with the shRNA control vector and shQSOX1 by electroporation, with conditions of 1100 mv, 20 pulses and 20 ms. Transfection was first confirmed by GFP images. Cells transfected with plasmids were selected with puromycin (4.5 μg/mL), and then survived cells were maintained in 0.1 μg/mL puromycin-containing media.

#### 4.5.2. Proliferation Assay

The same number of cells were seeded on 96-well plates and the cell number was measured by MTT (3-(4,5-dimethylthiazol-2-yl)-2,5-diphenyltetrazolium bromide) assay every 24 h.

#### 4.5.3. Measurement of Anti-Apoptotic Ability in Hypoxic Stress

LLC cells with the number of 2.0 × 10^5^ were seeded on 24-well plates. After 24 h, the medium was replaced with the complete medium containing H_2_O_2_ at the final indicated concentrations. After 8 h of treatment, toxicity and cell survival were assessed by MTT assays.

#### 4.5.4. Migration and Invasion Assay

Cell migration and invasion assays were performed in 24-well transwell plates (8-μm pore size, Corning, Corning, NY, USA). Matrigel (BD Bioscience, USA) was diluted to 1 mg/mL with serum-free culture medium and applied on the insert in the upper chambers of the multiwall for the invasion assay plate. Cells with the number of 1.0 × 10^5^ in 200 μL of the serum-free culture medium were seeded in the upper chambers of the wells. To induce chemotaxis of cells, 800 μL of 10% FBS medium was added to the lower chambers. After incubation for 24 h, at 37 °C, and 5% CO_2_, the membrane inserts were removed from the plate, and non-invading cells were removed from the upper surface of the membrane. Migrated or invaded cells were stained with 0.1% crystal violet for 20 min and washed with water. The invading cells were counted in at least 5 random fields using a microscope. The migrated cells were counted in 5 random fields by a cell confluence-measuring program in the JuLI FL microscope (NanoEntek, Seoul, Korea).

### 4.6. *In Vivo* Metastasis Assay in the Mouse Model

Seven- to eight-week-old C57BL/6 male mice were purchased from OrientBio (Daejeon, Korea). To establish the LLC mouse model, mice were i.v. injected with 1.5 × 10^6^ LLC cells through their tail vein. Mice were sacrificed 10 days and 32 days after tail vein injection, and lung, liver, and serum were collected for evaluation. All animal experiments were approved by the institutional animal care and use committee (IACUC) of Seoul National University. (SNU-131213-3)

### 4.7. Statistical Analysis

For statistical analysis, a *p*-value calculator for the student t-test and ANOVA was used. The results of each group were subjected to statistical analysis to assess differences compared to the control group. A *p*-value of less than 0.05 was considered to be significant.

Further information can be found in Supplementary Materials.

## 5. Conclusions

Here, our results proved that the secretome analysis of primary cancer and its stromal cells could find tissue-selective biomarkers. We found that QSOX1 can be a serum biomarker as detected by MRM and used in lung cancer diagnosis. In further study, evaluation of QSOX1 in a large number of samples might deduce more convincing evidence of QSOX1 as a biomarker of lung cancer. In addition, from its functions in promoting cancer metastasis, it might be further studied as a therapeutic target of lung cancer.

## Figures and Tables

**Table 1:** Proteins identified by LC–MS/MS analysis with an increase/decrease in lung cancer sample over 1.5-fold change.

IPI Number	Protein Name	20100622			20100719		
**Proteins Increased**		N **	LC **	**LC/N**	**N**	**LC**	**LC/N**
**IPI00328113**	FBN1 Fibrillin-1	5.55 *	74.58	13.44	1	4.31	4.31
**IPI00021405 (+2)**	LMNA Isoform A of Lamin-A/C	4.44	18.19	4.097	1	3.44	3.44
**IPI00292150**	LTBP2 Latent-transforming growth factor β-binding protein 2	3.33	13.64	4.096	1	2.58	2.58
**IPI00219219**	LGALS1 Galectin-1	4.44	12.73	2.867	1	3.44	3.44
**IPI00002714 (+2)**	DKK3 cDNA FLJ52545, highly similar to Dickkopf-related protein 3	7.77	20.92	2.692	1	5.17	5.17
**IPI00215965 (+3)**	HNRNPA1 Isoform A1-B of heterogeneous nuclear ribonucleoprotein A1	2.22	5.46	2.459	1	1.72	1.72
**IPI00000816**	YWHAE 14-3-3 protein epsilon	8.88	20.92	2.356	4.77	7.75	1.625
**IPI00008780**	STC2 Stanniocalcin-2	12.21	24.56	2.011	1	9.47	9.47
**IPI00032293**	CST3 Cystatin-C	8.88	17.28	1.946	3.58	6.89	1.925
**IPI00020977**	CTGF Isoform 1 of connective tissue growth factor	6.66	11.82	1.775	1	5.17	5.17
**IPI00216691**	PFN1 Profilin-1	8.88	15.46	1.741	2.38	6.89	2.895
**IPI00003351 (+1)**	ECM1 Isoform 1 of extracellular matrix protein 1	23.32	39.11	1.677	11.92	18.08	1.517
**IPI00003590**	QSOX1 Isoform 1 of sulfhydryl oxidase 1	5.55	9.1	1.64	1	4.31	4.31
**IPI00008556**	##IPI00008556	4.44	7.28	1.64	1	3.44	3.44
**IPI00003935 (+9)**	HIST2H2BE Histone H2B type 2-E	3.33	5.46	1.64	1	2.58	2.58
Proteins Decreased		**N**	**LC**	**LC/N**	**N**	**LC**	**LC/N**
**IPI00788247 (+1)**	KIF26A Kinesin-like protein KIF26A	2.22	1	0.45	2.38	1.72	0.723
**IPI00852669 (+1)**	ZNF516 Zinc finger protein 516	2.22	1	0.45	2.38	1.72	0.723
**IPI00008561 (+1)**	MMP1 Interstitial collagenase	11.1	3.64	0.328	17.88	8.61	0.482
**IPI00019223 (+4)**	AKAP9 Isoform 1 of A-kinase anchor protein 9	6.66	1	0.15	11.92	6.89	0.578

* Numbers in the table indicate relative peptide hits normalized by total ion current (TIC). ** N: normal, LC: lung cancer.

## Supplementary Materials

The following are available online at http://www.mdpi.com/1422-0067/19/10/3213/s1. Table S1: Patients’ clinical information, Table S2: Sequence and number of QSOX1 peptide identified by LC–MS/MS analysis, Table S3: Q1/Q2 Transitions for MRM experiments, Figure S1: Verification of QSOX1 increase in LLC mouse lung cancer model.
