# Supplementary material for: Quiescin Sulfhydryl Oxidase 1 (QSOX1) Secreted by Lung Cancer Cells Promotes Cancer Metastasis
Source: Int J Mol Sci. 2018 Oct 17;19(10):3213. doi: 10.3390/ijms19103213 (PMC6214099; doi:10.3390/ijms19103213)
Supplement: Supplementary file 1 [file ijms-19-03213-s001.zip › ijms-372413 supplementary/Supplementary Information_Sung et al.docx]

**Supplementary Information**

**Supplemental Methods**

**Sample preparation and MS methods for MRM**

*Serum depletion*

Before the in-solution digestion of serum, two high abundant serum proteins, albumin and IgG were removed from the crude serum. Depletion of two high abundant proteins was carried out by the protocols provided by manufacturer (Agilent Technologies, CA, USA). In short, 50ul of crude serum was diluted in 150ul of Buffer A. Diluted serum was applied to depletion column and flow through was collected and first wash fraction was combined.

*Instrument and LC/MRM-MS analysis*

The LC-MRM analysis has been conducted followed by previous report [^1^](#_ENREF_1).Depleted serum of healthy control, adenocarcinoma, and squamous cell carcinoma were subjected to in-solution tryptic digestion. Digested peptide mixture was desaltedby applying on to C18 spin column and then was dried. Pelleted peptides were dissolved in thirty microliters of 0.1% formic acid in HPLC water. One femtomole of a beta-galactosidase peptide (residues 293-299, LNVENPK, K heavy labeled) was added before the MS analysis as a relative internal standard peptide for quantitation. Peptides were separated using Agilent 1260 Infinity LC system with ChipCube Interface (Agilent, Santa Clara, CA). Separation was performed using binary gradients A (HPLC water in 0.1% formic acid solution) and B (Acetonitrile in 0.1% formic acid solution). The column was initially equilibrated and eluted at a flow rate of 0.4 μl/min for the nanopump and 4 μl/min for the capillary pump. The 30-minute gradient was programmed. Peptides were detected on an Agilent 6490 QQQ mass spectrometer. For the detection of QSOX1, appropriate peptide was selected for MRM analysis methods. The data were acquired by Skyline software (http://proteome.gs.washington.edu/software/skyline) in version 1.4.0.4421. Skyline was used for data extraction from the intensity chromatograms of the transitions and then highest (rank1) peak areas for transition was used quantitation whereas the remaining three transitions served as qualifiers and normalized abundant transition versus internal standard transitions (Q1/Q3 transitions at 411.2/594.3 m/z for the beta-galactosidase peptide).

**Western blot analysis**

Western blot analysis was performed according to previously reported procedures[^2^](#_ENREF_2). Briefly, 2.5µg of tissue proteins were separated and then detected with 1: 2,000 diluted QSOX1 antibody (polyclonal antibody to human QSOX1, ProteinTech Group Inc., Chicago, USA) followed by horseradish peroxidase-conjugated anti-rabbit IgG secondary antibodies (Thermo Scientific). Signals were developed with ECL-PLUS detection reagent (Thermo Scientific) and the membranes were then exposed to x-ray film for an appropriate time and then developed.

**Immunohistochemistry**

Immunohistochemistry experiments were performed as previously reported[^3^](#_ENREF_3). Following the procedure, QSOX1 antibody (polyclonal antibody to human QSOX1, ProteinTech Group Inc., Chicago, USA) at 1:50 dilution was applied to the samples of cancer tissues and their corresponding normal tissue microarray (ISU ABIXIS Co. Ltd, Seoul South Korea).

1. Ahn JM, Sung HJ, Yoon YH, et al. Integrated Glycoproteomics Demonstrates Fucosylated Serum Paraoxonase 1 Alterations in Small Cell Lung Cancer. Mol Cell Proteomics. 2013.

2. Kang SM, Sung HJ, Ahn JM, et al. The Haptoglobin beta chain as a supportive biomarker for human lung cancers. Mol Biosyst. 2011;7: 1167-1175.

3. Sung HJ, Ahn JM, Yoon YH, et al. Identification and validation of SAA as a potential lung cancer biomarker and its involvement in metastatic pathogenesis of lung cancer. J Proteome Res. 2011;10: 1383-1395.

**Supplemental Figure Legends**

**Supplementary FigureS1.Verification of QSOX1 increase in LLC mouse lung cancer model.** LLC cells were injected by i.v .and control mouse was injected with PBS only. First, one PBS injected mouse and two LLC cell injected mice were sacrificed after 10 days. No lung cancer nodules on lung were detected from both 2 mice. Then, after 35 days, 6 PBS injected mice and 7 LLC cell injected mice were sacrificed. All of the 7 LLC cell injected mice had lung cancer nodules on the lung and 2 of them had metastasis on liver and skin. (A) QSOX1 expression was confirmed on normal mouse lung (Group 1and 2), adjacent normal lung (N) and lung cancer nodules (T) or metastatic nodules of other organs (Meat-T) of LLC injected mouse lung (Group 3 and 4) (B).
