# Supplementary material for: Quiescin Sulfhydryl Oxidase 1 (QSOX1) Secreted by Lung Cancer Cells Promotes Cancer Metastasis
Source: Int J Mol Sci. 2018 Oct 17;19(10):3213. doi: 10.3390/ijms19103213 (PMC6214099; doi:10.3390/ijms19103213)
Supplement: Supplementary file 1 [file ijms-19-03213-s001.zip › ijms-372413 supplementary/Supplementary Tables_Sung et al.docx]

**Supplementary Table 1. Sequence and number of QSOX1 peptide identified by LC-MS/MS analysis**

| **Sample** | **Peptide sequence** | **Peptide hit** | |
| --- | --- | --- | --- |
|  |  | **Normal** | **Lung CA** |
| **20100622** | NNEEYLALIFEK | **1** | **2** |
|  | VLNTEANVVR | **1** | **2** |
|  | DFNIPGFPTVR | **3** | **5** |
|  | YSPSDPLTLLQADTVR | **0** | **1** |
|  | ***Spectrum count*** | ***5*** | ***10*** |
|  | ***Quantitative value*** | ***6*** | ***9*** |
| **20100719** | VPVLMESR | **0** | **1** |
|  | NNEEYLALIFEK | **0** | **4** |
|  | ***Spectrum count*** | ***0*** | ***5*** |
|  | ***Quantitative value*** | ***1*** | ***4*** |

**Supplementary Table 2. Q1/Q2 Transitions for MRM experiments**

| **Protein** | **Peptide sequence** | **Molecular**  **mass (Da)** | **Precursor ion**  **(*m/z*)** | **Transition**  **(*m/z*)** | **Fragment ion** | **CE**  **(V)** |
| --- | --- | --- | --- | --- | --- | --- |
| **QSOX1** | **VGSPNAAVLWLWSSHNR** | **1895.61** | **631.9955** | **899.4482** | **y7** | **18** |
|  |  |  |  | **786.3642** | **y6** | **18** |
|  |  |  |  | **600.2848** | **y5** | **18** |
|  |  |  |  | **513.2528** | **y4** | **18** |
| ***B-Gal*** | **LNVENPK** | **812.91** | **411.23** | **708.3766** | **y6** | **13.6** |
|  |  |  |  | **594.3337** | **y5** | **13.6** |
|  |  |  |  | **495.2653** | **y4** | **13.6** |

**Supplementary Table 3. Patients’ clinical information**

|  | **Serum samples** | | **Tissue samples** |
| --- | --- | --- | --- |
| **Type** | ***Healthy control*** | ***Lung cancer Patients*** | ***Lung cancer Patients*** |
| **Total Sample No.** | 30 | 60 | 62 |
| **Type**  **(Ad/SQ/Others)** | - | 30/30/0 | 40/21/1 |
| **Age Ave.**  **(Range)** | 55  (50-63) | 59.30  (50-65) | 63.90  (22-84) |
| **Sex**  **(M/F)** | 30/- | 60/- | 48/14 |
| **Smoking**  **(NS/SM/EX/ND)** | 5/12/13/0 | 3/45/7/5 | 17/23/20/2 |
| **Stages**  **(I/II/III/IV/UK)** | - | 18/4/12/21/5 | 40/14/6/0/- |

*Ages are given as average (range).

**Abbreviations used: Ad; Adenocarcinoma, SQ; squamous cell carcinoma, NS; non-smoker, SM; smoker, EX; ex-smoker, ND; non-described, UK; unknown
