# Supplementary figures and images for: Quiescin Sulfhydryl Oxidase 1 (QSOX1) Secreted by Lung Cancer Cells Promotes Cancer Metastasis
Source: Int J Mol Sci. 2018 Oct 17;19(10):3213. doi: 10.3390/ijms19103213 (PMC6214099; doi:10.3390/ijms19103213)

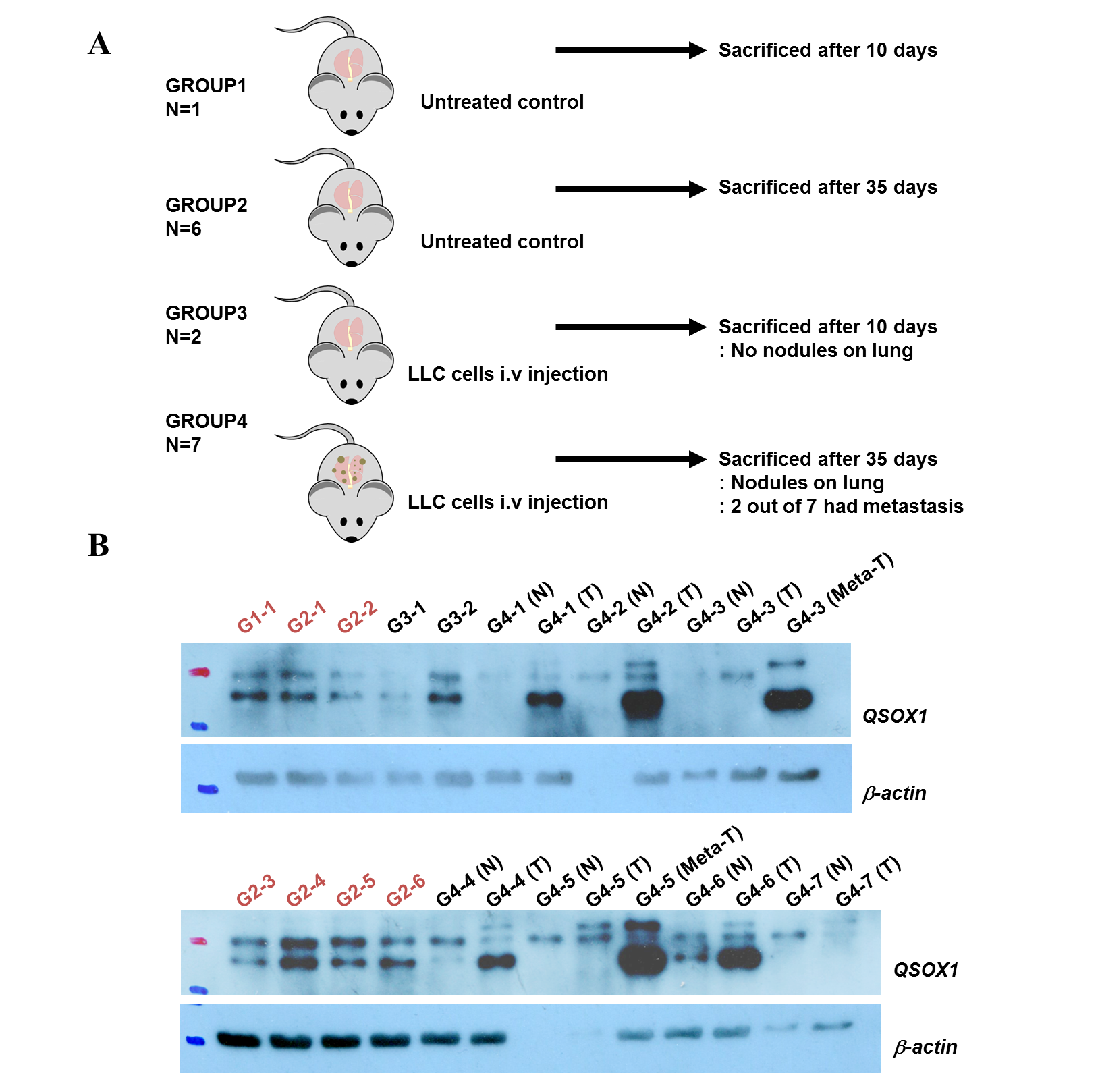

Supplement: Supplementary file 1 [file ijms-19-03213-s001.zip › ijms-372413 supplementary/Supplementary Figure 1.tif]
